# Hydronaphthol

**Published:** 1900-04-15

**Authors:** G. Monroe


### HYDRONAPHTHOL.

BY DR. G. MONROE.

Be careful to get hydronaphthol, not betanaphthol, as hydronaphthol is more soluble in water.

For cleansing a cavity previous to inserting a filling, use a solution of seven grains of hydronaphthol to an ounce of alcohol.

A solution of hydronaphthol in chloro-percha makes a good antiseptic dressing for root fillings. Hydronaphthol may also be mixed with oxychloride for root fillings.

As a dressing over sensitive dentin, use a hydronaphthol paste of oxychloride, with a couple of drops of oil of cloves or creosote.

A non-irritant cement may be made by mixing into oxyphosphate cement about one-fourth hydronaphthol.

For a mouth wash in pyorrhea alveolaris:

℞ Hydronaphthol, - - drams 2;
Tincture Calendula, - drams 4;
Distilled water, q.s.ad., ounces 8.

—*Dental Review.*

